# Factors Influencing 25‐Year Survival in Pediatric Liver Transplant Recipients

**DOI:** 10.1111/petr.70189

**Published:** 2025-09-25

**Authors:** Garrett Wortham, Megan Crawford, John Miggins, Chun‐Sing Huang, John A. Goss, Abbas Rana, Nhu Thao Galván

**Affiliations:** ^1^ Department of Student Affairs Baylor College of Medicine Houston Texas USA; ^2^ Division of Abdominal Transplant, Michael E DeBakey Department of Surgery Baylor College of Medicine Houston Texas USA

**Keywords:** biliary atresia, cholestatic liver disease, pediatric liver transplantation

## Abstract

**Background:**

This study assesses the impact of cholestatic liver disease, including biliary atresia, on 25‐year survival post‐transplantation and additional factors influencing long‐term outcomes after pediatric liver transplantation.

**Methods:**

We conducted a retrospective analysis of pediatric liver transplant recipients (1987–1998) using de‐identified data from the OPTN 2023 Liver Database. After exclusions for multi‐organ transplants (*n* = 222), prior transplants (*n* = 2256), and deaths within 1 year (*n* = 526), 2429 patients remained, including 645 with cholestatic disease. Univariate and multivariate analyses identified factors associated with 25‐year survival.

**Results:**

A primary diagnosis of cholestatic disease was associated with improved 25‐year survival (OR: 0.67, *p* = 0.023). UNOS regions 2 (OR: 0.65, *p* = 0.009) and 5 were also associated with improved survival (OR: 0.72, *p* = 0.044). Decreased survival was associated with recipient ages 8–12 (OR: 1.84, *p* = 0.009) and 12–18 (OR: 2.90, *p* = 0.003), donor age ≥ 19 (OR: 1.42, *p* = 0.021), African‐American (OR: 1.94, *p* = 0.000) and other minority recipient ethnicities (OR: 2.49, *p* = 0.018), donor bilirubin ≥ 2 mg/dL (OR: 1.85, *p* = 0.022), and UNOS region 10 (OR: 1.62, *p* = 0.009).

**Conclusion:**

This study evaluates 25‐year survival in pediatric liver transplant recipients while analyzing the protective influence of a cholestatic disease diagnosis. Older recipient and donor ages, minority recipient ethnicities, elevated donor bilirubin, and specific UNOS regions are associated with decreased 25‐year survival. Further research is needed to address outcome inequalities.

## Graphical abstract

Through a retrospective cohort analysis consisting of 2429 pediatric liver transplant recipients, we have identified variables influencing 25‐year survival. Notably, a recipient's primary diagnosis of cholestatic disease is protective for 25‐year survival.

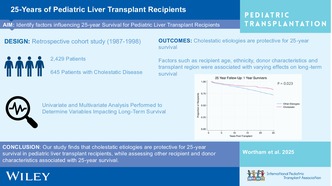

**Abbreviations**
BA: biliary atresia
GFR: glomerular filtration rate
ICU: intensive care unit
OPTN: organ procurement and transplantation network
PLTx: pediatric liver transplantation
UNOS: united network for organ sharing

## 1 Introduction

Pediatric patients with cholestatic liver disease, including biliary atresia (BA), are often in need of liver transplantation. The majority of pediatric patients listed for liver transplantation are younger than 1 year of age, with greater than 50% of patients being younger than 5 years of age [1, 2]. Among the various etiologies for pediatric cholestatic liver disease, biliary atresia remains the primary cause of obstructive cholestasis and is the leading indication for pediatric liver transplantation (PLTx), at 30% [1, 3]. Other etiologies of pediatric cholestatic liver disease include secondary biliary cirrhosis and primary sclerosing cholangitis.

Liver transplants in pediatric populations are generally associated with favorable long‐term outcomes. One study found that the 10‐year survival rate for PLTx recipients was 90%, while graft survival rates were 85% [4]. Previous literature has shown that there are several factors influencing long‐term survival following liver transplant, primarily recipient age, as younger recipients have demonstrated higher rates of 20‐year survival [5].

As pediatric patients have a long potential life expectancy, it is important to determine the factors that influence their long‐term survival post‐transplantation. This analysis evaluates factors that influence 25‐year survival in PLTx patients, including a primary diagnosis of cholestatic disease at the time of transplant listing.

## 2 Materials and Methods

### 2.1 Study Design

We retrospectively identified patients under the age of 18 listed for liver transplantation between 1987 and 1998 utilizing the Organ Procurement and Transplantation Network (OPTN) 2023 Liver Database. The United Network for Organ Sharing (UNOS) operates the Organ Procurement and Transplantation Network (OPTN). UNOS is primarily responsible for maintaining a central registry within the U.S. that matches organ donors and recipients, alongside the development of policies focused on the equitable allocation of organs. The OPTN database stores information relating to donors and recipients such as indications for listing, demographic information, and geographic location [6]. The public database contains de‐identified patient information, which exempts this study from IRB review.

### 2.2 Study Population

To identify 25‐year survival outcomes among pediatric patients, we only included liver transplants listed between October 16, 1987, and July 1, 1998, among patients who were below age 18 (*n* = 5433). Exclusions were made to control for confounding variables, including simultaneous multi‐organ transplant (*n* = 222), previous transplants and re‐transplants (*n* = 2256), and patient death within 1 year of transplantation (*n* = 526). We chose to exclude patients who died within 1 year of transplantation in order to accurately assess factors associated with only long‐term survival in our cohort while avoiding any confounding variables associated with short‐term mortality. This is an accepted method for controlling variables that influence short‐term mortality [7]. Our cohort, after exclusions were made, included 2429 patients. Demographic data is included in Table 1.

**TABLE 1:** This table depicts the study cohort donor and recipient demographics.

Total recipients	Survival < 25 years	Survival > 25 years	*p*
Total	442	1987	n/a
Recipient demographics and characteristics			
Recipient age	6.751131 ± 2.01e‐16	4.212884 ± 6.03e‐16	0.000
Weight (kg)	27.29085 ± 3.04e‐14	18.69056 ± 4.52e‐15	0.000
Diagnosis: cholestatic	80 (12.40%)	565 (87.60%)	0.000
Albumin (mg/dL)	3.128293 (2.51e‐15)	3.238735 (4.95e‐15)	0.009
Creatinine	0.565358 ± 4.23e‐16	0.4478729 ± 1.79e‐15	0.001
Hemodialysis at transplant	4 (16.67%)	20 (83.33%)	0.845
Intensive care at transplant	120 (20.48%)	466 (79.52%)	0.101
Mechanical support at time of transplant	69 (22.19%)	242 (77.81%)	0.051
Recipient total bilirubin	12.79516 ± 9.41e‐15	12.08088 ± 2.57e‐14	0.251
Ascites	442 (18.20%)	1987 (81.80%)	1
Body mass index	18.87173 ± 1.11e‐14	18.17109 ± 2.50e‐14	0.181
African–American recipient	117 (28.82%)	289 (71.18%)	0.000
Ventilation at transplant	66 (22.60%)	226 (77.40%)	0.038
Private insurance	101 (15.63%)	545 (84.37%)	0.049
Previous abdominal surgery	78 (13.54%)	498 (86.46%)	0.001
Donor and allograft demographics and characteristics			
Donor age	15.35601 ± 3.75e‐16	10.75843 ± 1.56e‐17	0.000
African–American donor	64 (20.58%)	249 (79.42%)	0.244
Live donor transplant	28 (14.36%)	167 (85.64%)	0.149
Split transplant	82 (16.24%)	423 (83.76%)	0.200
Cold ischemia time (hours)	10.07217 ± 9.91e‐16	10.61329 ± 9.90e‐16	0.109
Donor total bilirubin	1.066486 ± 3.89e‐16	0.9325826 ± 8.91e‐16	0.402
Donor: diabetes mellitus	186 (18.22%)	835 (81.78%)	0.770

### 2.3 Statistical Analysis

Data were analyzed utilizing Stata 18.0 (Stata Corp, College Station, TX, USA). Continuous variables were reported as mean ± standard deviation and compared using the Student's *t*‐test. We utilized contingency table analysis to compare categorical variables. Results were considered significant at *p* < 0.05. Kaplan–Meier survival analysis was utilized to estimate survival over time.

Univariable linear regression analysis was performed on variables included in the PEDI‐SOFT Liver score, as well as a recipient diagnosis of cholestatic disease. Recipient diagnoses of cholestatic disease were identified utilizing the diagnosis codes for cholestatic liver disease/cirrhosis and biliary atresia, as defined by the OPTN. Significant factors in univariable analysis were included in a multivariable linear regression analysis.

### 2.4 Risk Factors

The recipient risk factors considered were age, weight (kg), primary diagnosis, ethnicity, albumin, creatinine, hemodialysis status, ABO blood type, intensive care unit (ICU) stay, mechanical support status, glomerular filtration rate (GFR), transplant type, total bilirubin, ascites, BMI, ventilation status, private insurance, previous abdominal surgery, recipient urgency status of 1, and transplant region. We used absolute recipient BMI values instead of z‐scores because our patient population consists of recipients with cholestatic etiologies, who often have lower‐than‐average BMIs. Since z‐scores are relative to the general public, they may have been more difficult to interpret in this analysis.

The donor risk factors considered were age, ethnicity, live donor transplant, split transplant, whole transplant, cold ischemia time, total bilirubin, cause of death, diagnosis of diabetes mellitus, and ABO compatibility with recipient.

The Schwartz bedside formula: eGFR = 0.41 × height (cm)/Scr (mg/dL) was used for the calculation of creatinine clearance. Serum sodium, Pediatric End‐Stage Liver Disease scores, and Model for End‐Stage Liver Disease scores were not consistently recorded before 2002 and are excluded from our analysis.

## 3 Results

### 3.1 Study Population

Our study population included 2429 patients after appropriate exclusions were made. Demographic and clinical characteristics are displayed in Table 1. Significant recipient demographics include recipient age, weight (kg), a primary diagnosis of cholestatic disease, albumin (mg/dL), creatinine, African American ethnicity, ventilation at the time of transplant, private insurance, and previous abdominal surgery. The one significant donor demographic is donor age.

**FIGURE 1:**
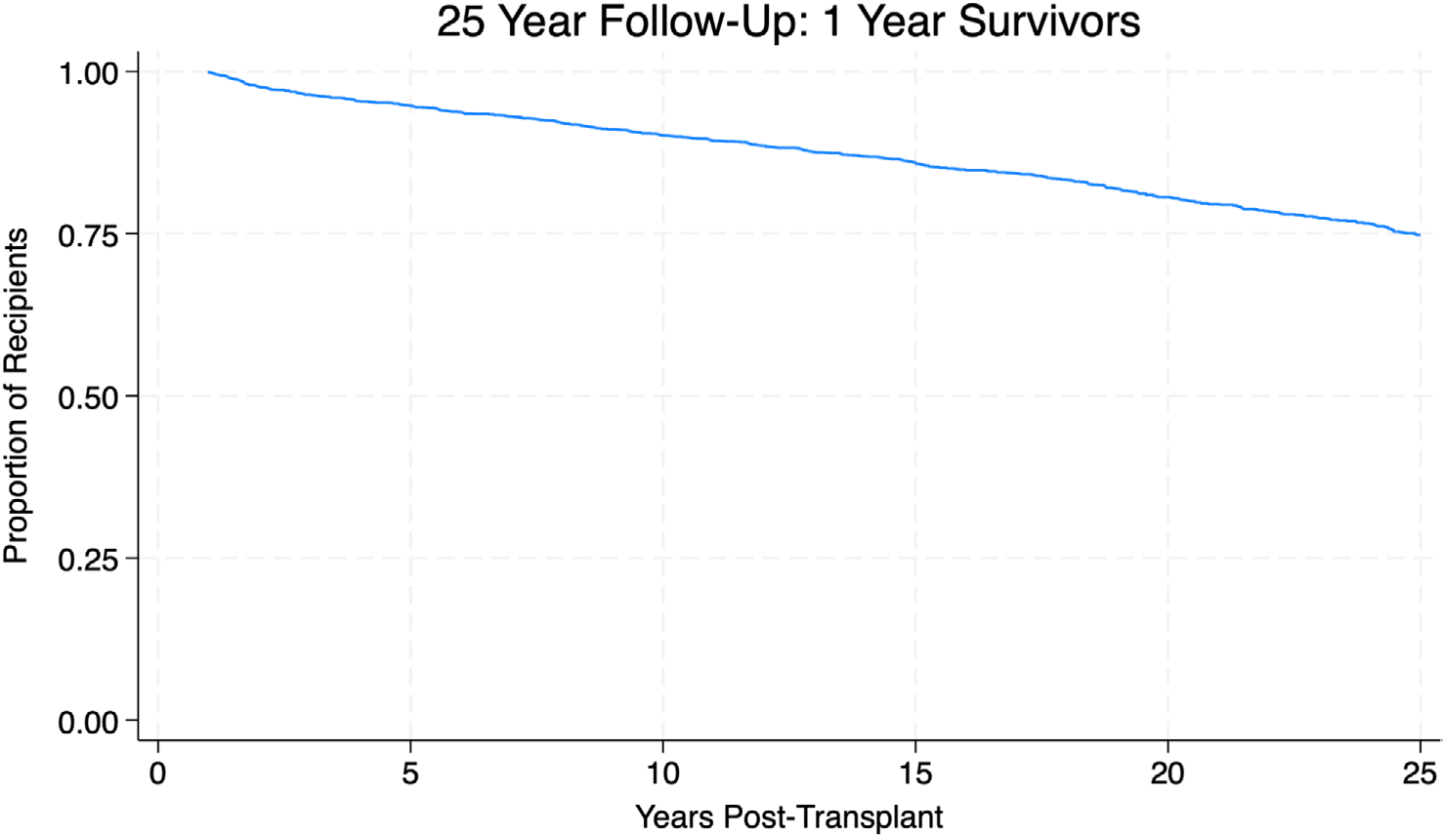
This figure depicts a 25‐year survival Kaplan–Meier curve in the study cohort that survived beyond 1 year post‐transplantation.

**FIGURE 2:**
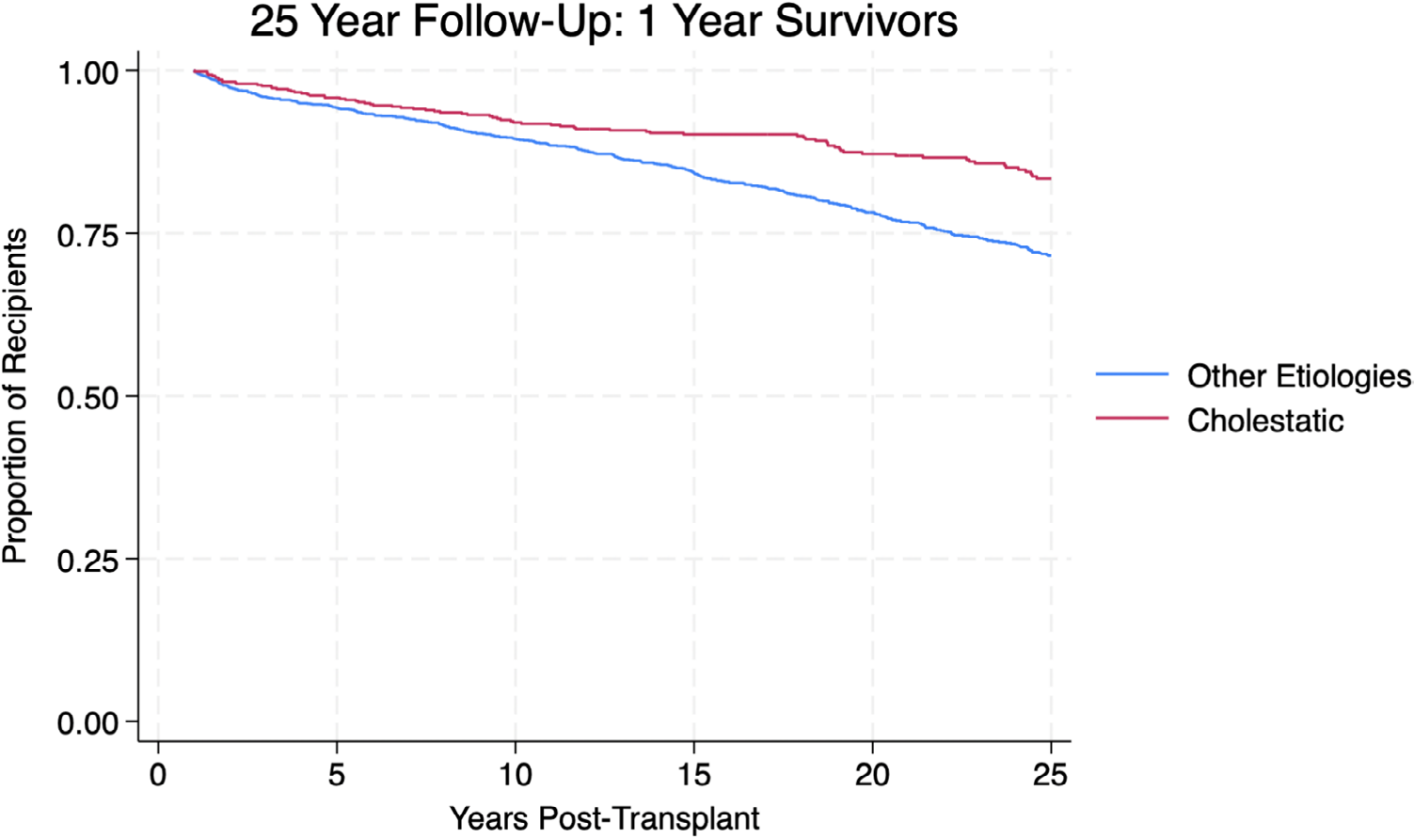
This figure depicts a 25‐year survival Kaplan–Meier curve comparing cholestatic etiologies versus all other recipient etiologies.

Figure 1 illustrates a 25‐year survival Kaplan–Meier curve of the study cohort. Figure 2 illustrates a Kaplan–Meier curve representing the difference in 25‐year survival between a primary diagnosis of cholestatic disease and all other etiologies. Significant variables for 25‐year survival in multivariable analysis were a primary recipient diagnosis of cholestatic disease, African‐American recipient ethnicity, other recipient ethnicities (not including Hispanic, White, African‐American, or Asian), elevated donor bilirubin, donor age ≥ 19, transplants within regions 2, 5, and 10, recipient ages 8–12, and recipient ages 12–18.

### 3.2 Data Entry Rate

Table 2 displays the entry rates for the variables considered in the analysis. Most variables had an entry rate above 90%.

**TABLE 2:** This table displays the multivariate logistic regression for factors that predict 25‐year survival in 1‐year survivors.

Variable	Entry completion (%)	OR	*p*
Cholestatic diagnosis	100%	0.6685286	0.023
Recipient age < 1	100%	0.8229396	0.217
Recipient age 8–12	100%	1.839236	0.009
Recipient age 12–18	100%	2.897598	0.003
Recipient age 10–15	100%	0.7199355	0.278
Recipient age 15–18	100%	0.544693	0.120
Region 2	100%	0.6549692	0.009
Region 5	100%	0.7223795	0.044
Region 10	100%	1.617579	0.009
Elevated donor bilirubin (≥ 2 to < 90 mg/dL)	41.25%	1.854303	0.022
African–American recipient	100%	1.93941	0.000
Other ethnicity recipient	100%	2.494407	0.018
Previous abdominal surgery	99.79%	0.930061	0.681
Private health insurance	50.39%	0.7633485	0.055
White recipient	100%	0.9157406	0.581
Body mass index < 18.5	83.74%	0.8381697	0.235
Body mass index ≥ 18.5 to < 25	83.74%	0.9033023	0.577
Body mass index ≥ 25 to < 30	83.74%	1.545369	0.138
Body mass index ≥ 30 to < 35	83.74%	1.817664	0.194
Distance ≥ 250 miles	97.90%	0.9460659	0.664
Cold ischemia time < 6 h	91.19%	1.223509	0.162
Creatinine 1.5–2.0	98.15%	1.770295	0.228
Recipient weight > 6 to ≤ 7 kg	99.34%	0.8717653	0.562
Recipient weight > 9 to ≤ 10 kg	99.34%	0.6917814	0.169
Donor age < 1	99.96%	0.9743715	0.904
Donor age 1–2	99.96%	0.9739028	0.883
Donor age 4–5	99.96%	0.6538807	0.196
Donor age 12–18	99.96%	1.215548	0.308
Donor age ≥ 19	99.96%	1.419506	0.021
Ventilation at time of transplant	100%	1.116643	0.510

### 3.3 Primary Recipient Diagnosis

In our multivariate analysis, a diagnosis of cholestatic disease was found to be protective for 25‐year survival post‐transplantation (OR: 0.67, *p* = 0.023). Figure 2 illustrates the difference in 25‐year survival between cholestatic etiologies and all other etiologies.

### 3.4 Recipient Ethnicity

African‐American ethnicity, as well as other ethnicities (all ethnicities excluding Hispanic, White, African‐American, or Asian), is considered a significant risk factor for 25‐year survival post‐transplantation. African‐American recipients have a significantly higher risk of mortality post‐transplant, as seen in multivariable analysis (OR = 1.94, *p* = 0.000). Other ethnicities, which include all ethnicities listed on the United Network for Organ Sharing (UNOS) database other than Hispanic, White, African‐American, or Asian, also have an increased risk of mortality post‐transplant when analyzed in multivariable analysis (OR = 2.49, *p* = 0.018).

### 3.5 Recipient Region

Regions 2, 5, and 10, as defined by UNOS, were considered significant in our multivariable analysis. Regions 2 and 5 were found to be protective for 25‐year survival post‐transplantation, while region 10 was found to be a risk factor for 25‐year survival post‐transplantation. Region 2 (consisting of Delaware, the District of Columbia, Maryland, New Jersey, Pennsylvania, and West Virginia) was found to be protective for 25‐year survival (OR = 0.65, *p* = 0.009). Region 5 (consisting of Arizona, California, Nevada, New Mexico, and Utah) was also found to be protective for 25‐year survival (OR = 0.72, *p* = 0.044). Region 10 (consisting of Indiana, Michigan, and Ohio), on the other hand, was found to be a risk factor for 25‐year survival (OR = 1.62, *p* = 0.009).

### 3.6 Recipient Age

In our multivariable analysis, older recipient age was found to be a significant risk factor for 25‐year survival post‐transplantation. Recipients aged 8–12 had an increased risk for mortality post‐transplantation (OR = 1.84, *p* = 0.009). Recipients aged 12–18 also had an elevated risk for mortality post‐transplantation (OR = 2.90, *p* = 0.003).

### 3.7 Donor Bilirubin

Elevated donor bilirubin (2–90 mg/dL) was found to be a risk factor for 25‐year survival post‐transplantation in multivariable analysis (OR = 1.85, *p* = 0.022).

### 3.8 Donor Age

Older donor age (≥ 19 years of age) was found to be a risk factor for 25‐year survival post‐transplantation in multivariate analysis (OR = 1.42, *p* = 0.021).

## 4 Supplementary Analysis

### 4.1 Supporting Information: Analysis of Graft Failure

We analyzed graft failure trends within our cohort, with graft failure being defined as the allograft surviving less than 25 years post‐transplant date. Primary diagnosis of cholestatic disease was not found to be significant in terms of graft failure. Factors found to be protective in our multivariate analysis include regionality, specifically regions 2 and 5, as well as regional transplants. Risk factors found within our multivariate analysis include region 10, African‐American recipients, and recipients who did not identify with the UNOS ethnicity categories (White, Hispanic, African‐American, or Asian). The multivariate logistic regression for graft failure is summarized in Table S1, and the Kaplan–Meier survival curve for this analysis is depicted in Figure S1.

### 4.2 Supporting Information: Protective Factors and Risk Factors Associated With 25‐Year Survival Within Patients Diagnosed With Cholestatic Disease

Alongside our initial study cohort, we analyzed factors that influenced 25‐year survival exclusively in cholestatic patients. We utilized the same exclusions as the original patient cohort, with the addition of excluding any patients not diagnosed with a cholestatic etiology. This created a cohort of 645 patients. In our multivariate analysis, protective factors for 25‐year survival include previous abdominal surgery and a recipient urgency status of 1. Risk factors against 25‐year survival include a recipient glomerular filtration rate (GFR) of 10–20 mL/min, region 10, and donor age above or equal to 19. The multivariate logistic regression for graft failure is summarized in Table S2, and the Kaplan–Meier survival curve for this analysis is depicted in Figure S2.

## 5 Discussion

Our results indicate that factors promoting long‐term 25‐year PLTx recipient survival include a primary recipient diagnosis of cholestatic disease and regionality, specifically regions 2 and 5. Table 2 displays the odds ratio and *p*‐value for each of these variables.

Risk factors hindering long‐term PLTx recipient survivability include older recipient age (ages 8–12 and 12–18), transplants occurring within region 10, elevated donor bilirubin levels, African‐American recipient ethnicity, alongside other minority ethnicity recipients, and a donor age above or equal to 19. Table 2 illustrates the odds ratio and *p*‐value for these variables.

### 5.1 Primary Diagnosis of Cholestatic Disease

Within the adult population, cholestatic liver disease (including primary sclerosing cholangitis and primary biliary cholangitis) has remarkably good transplant outcomes compared to other etiologies necessitating liver transplantation [8, 9]. In regard to pediatric patients, biliary atresia is the primary indication for liver transplantation and is associated with excellent patient and graft survival [10]. Within our study cohort of 2429 patients, 645 patients had a primary diagnosis of cholestatic disease. Of these patients, 510 were diagnosed with biliary atresia, meaning approximately 79% of patients with cholestatic etiologies had a primary diagnosis of biliary atresia. Our results are consistent with previous literature regarding the protective nature of cholestatic etiologies, the majority of which is composed of biliary atresia, as an indication for liver transplantation. Figure 2 displays the Kaplan–Meier survival curve showing the protective nature of cholestatic disease versus all other etiologies, illustrating the discrepancy in long‐term survival between cholestatic etiologies and other etiologies. This information can assist health‐care providers in providing prognostic counseling for their patients, as biliary atresia is a significant indication for liver transplantation within the pediatric population.

There are several mechanisms that could assist in explaining why cholestatic etiologies, primarily biliary atresia, are protective for long‐term survival. Biliary atresia, while fatal if left untreated, is a fibroinflammatory cholangiopathy that is primarily isolated to the biliary system. One multicenter prospective study found that 84% of pediatric patients with biliary atresia had non‐syndromic disease without involvement of other major organ systems [11]. These favorable outcomes post‐transplant can likely be attributed to biliary atresia's non‐syndromic presentation. Additionally, patients with biliary atresia tend to have preserved renal function both pre‐transplant and post‐transplant [12, 13, 14]. These mechanisms, alongside more general advancements in the perioperative management of pediatric liver transplant recipients, may assist in explaining the protective nature of cholestatic etiologies versus all other etiologies for 25‐year survival.

### 5.2 Recipient Ethnicity

African‐American recipient ethnicity, as well as recipients that did not identify with the UNOS ethnicity categories (White, Hispanic, African‐American, or Asian), was found to be a risk factor against long‐term survival post‐liver transplant in our multivariate analysis. Whether racial disparities in post‐transplant outcomes affect pediatric patients has been a point of contention in previous literature, with some studies suggesting that the disparities in outcomes only exist among adult populations [15]. Recent literature, however, suggests that pediatric African‐American liver transplant recipients have larger rates of mortality compared to other ethnicities [5]. Previous literature has also found that among PLTx recipients transferred to adult care, African‐American patients have higher mortality rates, suggesting that African‐American patients are not only disproportionately affected in the post‐operative period but throughout their entire lifetimes [16]. Our research supports the notion that African‐American (as well as a listing of “other ethnicity”) pediatric liver transplant recipients have higher rates of mortality and poor long‐term 25‐year survival rates compared to other ethnicities. There are likely several systemic reasons for this large discrepancy in transplant outcomes based on race. For example, African‐American patients are less likely to be referred for liver transplantation and are typically referred for transplantation at later stages of disease severity [17]. Studies have found the incidence of biliary atresia to be increasing, with African‐American recipients having a greater incidence of biliary atresia compared to White recipients [18]. This increasing incidence highlights the need to find methods in which we can reduce the survival disparity seen among African‐American recipients.

In recent years, African‐American patients have seen improved post‐transplant survival rates. One retrospective cohort analysis using the UNOS database found a significant decrease in post‐transplant mortality among African‐American recipients within the last decade; however, similar reductions in mortality were found in other races as well, suggesting that discrepancies in outcomes still exist for African‐American recipients compared to other recipient ethnicities [19]. Improvement in PLTx survival rates may be partially attributable to national and state policy changes, such as Medicaid expansion, which has been shown to decrease the risk of poor outcomes in African‐American PLTx recipients by over 10% [20]. While recent literature suggests improvement in outcomes for African‐American PLTx recipients, there remains a significant discrepancy in outcomes when compared to other recipient ethnicities. These findings are likely attributable to decades of systemic inequities in healthcare and provide a strong justification for continued policy work and legislation aimed at delivering equitable care for historically marginalized and vulnerable populations.

### 5.3 Recipient Region

Regions 2 and 5 were also found to be protective factors associated with 25‐year survival within our multivariate analysis, while region 10 was found to be associated with increased risk. These regions are defined by the United Network for Organ Sharing. While the literature is scarce regarding UNOS regionality influences on post‐transplant morbidity, literature conducted between 2010 and 2015 suggests discrepancies in waitlist mortality among the different regions, primarily region 5 [21]. One proposed explanation for why there is poor long‐term survival in region 10 compared to regions 2 and 5 is a lack of live donor transplants. Live donor transplants had better outcomes during this era of study, and a lack of these alternative graft sources could potentially explain the poor survival seen in region 10 [22].

Additionally, previous studies have shown that low‐volume transplant centers have poorer post‐transplant outcomes compared to higher‐volume transplant centers, as low‐volume pediatric transplant centers tend to be more adult‐care focused, with fewer resources than higher‐volume centers [23, 24]. In previous literature, low‐volume pediatric centers have been defined as fewer than 5 transplants performed annually. One large cohort study consisting of 6628 pediatric liver transplant recipients found that in these low‐volume centers (with the criteria being fewer than 5 transplants performed annually), only 41% of patients received a transplant, compared to 85% in centers with higher volume [23]. More of these low‐volume pediatric centers may be located within UNOS region 10 than UNOS regions 2 and 5 and could provide a possible correlation to poorer long‐term outcomes in the region. Recent literature has focused on the restructuring of UNOS regions to decrease outcome discrepancies. One study found that the replacement of traditional donor service areas with large fixed‐distance circles surrounding the donor hospital (400 miles) could lead to greater equitable donor supply and recipient demand ratios [25]. The continuous discussion regarding ways in which we can reduce disparities between UNOS regions should be used to influence the restructuring of current regional systems. Additionally, further research is warranted to determine the number of high‐and low‐volume centers within each UNOS region, as this may assist in explaining regional differences in outcomes.

### 5.4 Recipient Age

Older recipient age (age groupings 8–12 and 12–18) was found to be a risk factor against 25‐year survival in our multivariate analysis. It has been documented that younger recipient ages at the time of liver transplant correspond to greater 20‐year survival rates [5]. Our research corresponds with this finding, within the scope of 25‐year survival. Rationale as to why older patients typically have worse outcomes is multifactorial and includes medication nonadherence, complications from immunosuppression, and challenges with the transition from pediatric to adult care.

Medication nonadherence is the most common cause of late acute rejection among PLTx patients and ultimately leads to higher rates of mortality [26]. It has been estimated that nonadherence to immunosuppressive medications occurs in 35% to 50% of adolescents and may be a contributing factor to the comparatively poor long‐term outcomes seen in this age group after transplantation [26].

Additionally, older recipient age is associated with greater rates of complications from immunosuppression treatment. Immunosuppressive therapy, particularly the usage of calcineurin inhibitors, has been shown to cause nephrotoxicity, with older recipient age at the time of transplant being a risk factor for nephrotoxicity [27]. These increased rates of complications from immunosuppressive therapy may assist in explaining the decreased percentage of long‐term survival seen in older recipients.

Older recipients also face the challenging transition from pediatric to adult care. This transition to adult services is often based on chronological age, without considering the recipients' preparedness for the transition, and may confer challenges to medical adherence that negatively influence outcomes [27]. Recent literature has emphasized the importance of a standardized approach to this transition of care, with the development of a structured framework for this transition outlined by the North American Society of Pediatric Gastroenterology, Hepatology, and Nutrition [28]. Implementation of frameworks such as these between transplant centers may alleviate the poor outcomes traditionally seen within this vulnerable age group.

### 5.5 Donor Bilirubin

Elevated donor bilirubin (≥ 2 to < 90 mg/dL) was found to be a risk factor against 25‐year survival in multivariate analysis. Previous literature has shown that grafts with hyperbilirubinemia are oftentimes not utilized, but that favorable post‐transplant outcomes in adults can still be achieved with these grafts, leading to greater graft availability for recipients [29, 30]. Despite the potential to utilize grafts with hyperbilirubinemia, it remains important to make a comprehensive assessment based on the recipient's urgency status and the availability of other grafts.

Hyperbilirubinemia in pediatric patients is a proposed mechanism of cirrhotic cardiomyopathy, in which there is both systolic and diastolic cardiac dysfunction in the absence of other cardiac disease, and is associated with increased multiorgan dysfunction and complications post‐transplant [31, 32]. Pediatric patients with BA already have excess levels of bile acids, which are associated with pathological remodeling of the heart (specifically pathologic alterations in left ventricular geometry), and donor grafts utilized from donors with excess bilirubin may exacerbate this cardiomyopathy [33]. Recent studies have shown that reduction of bile acid concentrations (either through albumin‐charcoal dialysis or plasma exchange) can potentially provide a therapeutic means of reducing cardiomyopathy in these patients [33]. With the reduction of cardiomyopathy in these patients, complications due to the utilization of grafts with hyperbilirubinemia can be limited, possibly leading to better long‐term outcomes for these recipients. Further research is needed to identify instances in which elevated donor bilirubin allografts can be safely implemented in pediatric transplantation.

### 5.6 Donor Age

Within our research, we found that there is a significant decrease in 25‐year survival among recipients who receive grafts from donors at or above the age of 19. This finding parallels previous literature that finds that grafts from older donors contribute to an elevated risk of mortality [34, 35, 36]. Recent studies have suggested that although younger donor age demonstrates survival benefits in recipients post‐transplant (especially in recipients aged 0–2), a greater emphasis should be placed on a quick turnaround from waitlist listing to transplant date, rather than the specific characteristics of the donor graft [37]. This demonstrates the need for healthcare providers to balance the beneficial attributes of an “optimal” graft versus the impact of time on the waitlist for recipients. Further discussion on this topic is necessary, focusing on how we can efficiently match recipients and donors for the best overall outcomes.

### 5.7 Limitations

Limitations of our study include the inability to account for recent advancements within the field of pediatric liver transplantation, due to analyzing transplants occurring between 1987 and 1998. Modern changes in surgical techniques and post‐operative management may have an influence on variables impacting long‐term survival in these patients.

As with other large registries, the UNOS database is susceptible to inconsistencies in data entry. Our study aims to mitigate this limitation through the utilization of variables with a high entry completion percentage; however, some variables have relatively low entry rates.

## 6 Conclusion

Our study finds that a primary diagnosis of cholestatic disease is protective of 25‐year survival in PLTx patients. Other protective factors associated with 25‐year survival in PLTx patients include regionality, specifically regions 2 and 5.

Risk factors associated with 25‐year survival in PLTx patients include older recipient age, transplants within region 10, elevated donor bilirubin levels, older donor age, and African‐American recipient ethnicity as well as other minority ethnicity recipients.

With biliary atresia being the leading indication for pediatric liver transplantation, the high rates of long‐term survival seen in this patient population can be utilized in prognostic counseling for patients and their families. Both medical practitioners and policymakers must consider the various discrepancies that currently influence long‐term survival post‐pediatric liver transplantation, including regionality and racial disparities, and create guidelines and legislation aimed at reducing these disparities. Recent literature has placed a greater emphasis on the equitable restructuring of existing UNOS regions, alongside methods to reduce racial inequities in outcomes post‐transplant, which may assist in reducing the poor prognostic influence of these variables in the modern age of transplantation.

## Disclosure

Senior Author Dr. Nhu Thao Galván has received a Natera grant from ASTS. This grant has no influence on the content of this manuscript.

## Supporting information


**Data S1.** petr70189‐sup‐0001‐AppendixS1.docx.

## Data Availability

The data that support the findings of this study are openly available in OPTN at https://optn.transplant.hrsa.gov/data/view‐data‐reports/request‐data/.
