# Supplementary material for: Factors Influencing 25‐Year Survival in Pediatric Liver Transplant Recipients
Source: Pediatr Transplant. 2025 Sep 25;29(7):e70189. doi: 10.1111/petr.70189 (PMC12464467; doi:10.1111/petr.70189)
Supplement: Supplementary file 1 — Data S1. petr70189‐sup‐0001‐AppendixS1.docx. [file PETR-29-e70189-s001.docx]

### 1. Supplementary Data

#### 1.1 Supplementary Data: Analysis of Graft Failure

We analyzed graft failure trends within our cohort, with graft failure being defined as the allograft surviving less than 25 years post-transplant date. Primary diagnosis of cholestatic disease was not found to be significant in terms of graft failure. Factors found to be protective in our multivariate analysis include regionality, specifically regions 2 and 5, as well as regional transplants. Risk factors found within our multivariate analysis include region 10, African-American recipients, and recipients that did not identify with the UNOS ethnicity categories (White, Hispanic, African-American, or Asian). The multivariate logistic regression for graft failure is summarized in Table S1, and the Kaplan-Meier survival curve for this analysis is depicted in Figure S1.

#### 1.2 Supplementary Data: Protective Factors and Risk Factors associated with 25-year survival within patients diagnosed with cholestatic disease

Alongside our initial study cohort, we analyzed factors that influenced 25-year survival exclusively in cholestatic patients. We utilized the same exclusions as the original patient cohort, with the addition of excluding any patients not diagnosed with a cholestatic etiology. This created a cohort of 645 patients. In our multivariate analysis, protective factors for 25-year survival include previous abdominal surgery and a recipient urgency status of 1. Risk factors against 25-year survival include a recipient glomerular filtration rate (GFR) of 10-20 mL/min, region 10, and donor age above or equal to 19. The multivariate logistic regression for graft failure is summarized in Table S2, and the Kaplan-Meier survival curve for this analysis is depicted in Figure S2.

### Supplementary Tables

**Table S1.** This table displays the multivariate logistic regression for factors that predict 25-year graft survival.

| **Variable** | **Entry completion (%)** | **OR** | **p-value** |
| --- | --- | --- | --- |
| Cholestatic Diagnosis | 100% | .7138279 | .063 |
| Recipient Age < 1 | 100% | .8731942 | .384 |
| Recipient Age 10-15 | 100% | 1.066086 | .802 |
| Recipient Age 15-18 | 100% | .9228804 | .825 |
| Recipient Age 12-18 | 100% | 1.607115 | .108 |
| Donor Age 1-2 | 99.96% | .988016 | .943 |
| Donor Age ≥ 19 | 99.96% | 1.230879 | .150 |
| **Region 2** | **100%** | **.5770904** | **.002** |
| Region 3 | 100% | 1.293812 | .214 |
| **Region 5** | **100%** | **.7032467** | **.039** |
| **Region 10** | **100%** | **1.66695** | **.007** |
| **Regional Transplant** | **100%** | **.7223564** | **.020** |
| Mechanical Support | 100% | 1.180498 | .323 |
| Cold ischemia time <6 hrs | 91.19% | 1.285942 | .091 |
| Recipient Glomerular Filtration Rate 10-20 mL/min | 100% | 2.631075 | .088 |
| Distance ≥ 250 miles | 100% | .9545588 | .724 |
| Body Mass Index < 18.5 | 83.74% | .8629094 | .238 |
| Body Mass Index ≥ 25 - < 30 | 83.74% | 1.472511 | .173 |
| White recipient | 100% | .8169894 | .213 |
| **African-American recipient** | **100%** | **1.71131** | **.004** |
| **Other ethnicity recipient** | **100%** | **2.915915** | **.006** |
| Previous abdominal surgery | 99.79% | .8490484 | .374 |
| Recipient Weight > 6 kg - ≤ 7 kg | 99.34% | .8631173 | .546 |
| Height difference ≥ -60 & < -30 | 100% | .7505418 | .217 |
| Cause of death: Cerebrovascular Accident | 100% | 1.17942 | .307 |
| Creatinine 1.5-2.0 | 98.15% | 1.908936 | .170 |

**Table S2.** This table displays a multivariate logistic regression for factors that predict 25-year survival in cholestatic patients.

| **Variable** | **Entry completion (%)** | **OR** | **p-value** |
| --- | --- | --- | --- |
| Recipient Body Mass Index <18.5 | 100% | .7343551 | .399 |
| Recipient Body Mass Index ≥ 18.5 - < 25 | 100% | 1.516021 | .335 |
| Years on waitlist >= 1 and < 2 | 100% | 1.712611 | .213 |
| Distance ≥ 250 miles | 100% | .8927214 | .735 |
| **Recipient Glomerular Filtration Rate 10-20 mL/min** | **100%** | **11.29264** | **.011** |
| **Region 10** | **100%** | **2.247617** | **.030** |
| **Recipient Urgency Status of 1** | **100%** | **.3213515** | **.004** |
| Recipient Weight > 6 kg - ≤ 7 kg | 100% | .6486892 | .363 |
| Recipient Age < 1 | 100% | .677556 | .244 |
| Recipient Age 10-15 | 100% | 1.272842 | .679 |
| Recipient Age 15-18 | 100% | 1.387971 | .734 |
| Recipient Age 12-18 | 100% | 2.206062 | .259 |
| Donor Age 1-2 | 100% | .5978586 | .241 |
| Donor Age 10-15 | 100% | 1.103192 | .855 |
| Donor Age 12-18 | 100% | 1.555124 | .348 |
| **Donor Age ≥ 19** | **100%** | **2.101456** | **.026** |
| **Previous Abdominal Surgery** | **100%** | **.4938573** | **.009** |
| African-American Recipient | 100% | 1.606701 | .122 |

Supplementary Figures

**Figure S1.** This figure illustrates a 25-year Kaplan-Meier Survival curve comparing cholestatic etiologies vs. all other recipient etiologies in the context of graft failure.

**
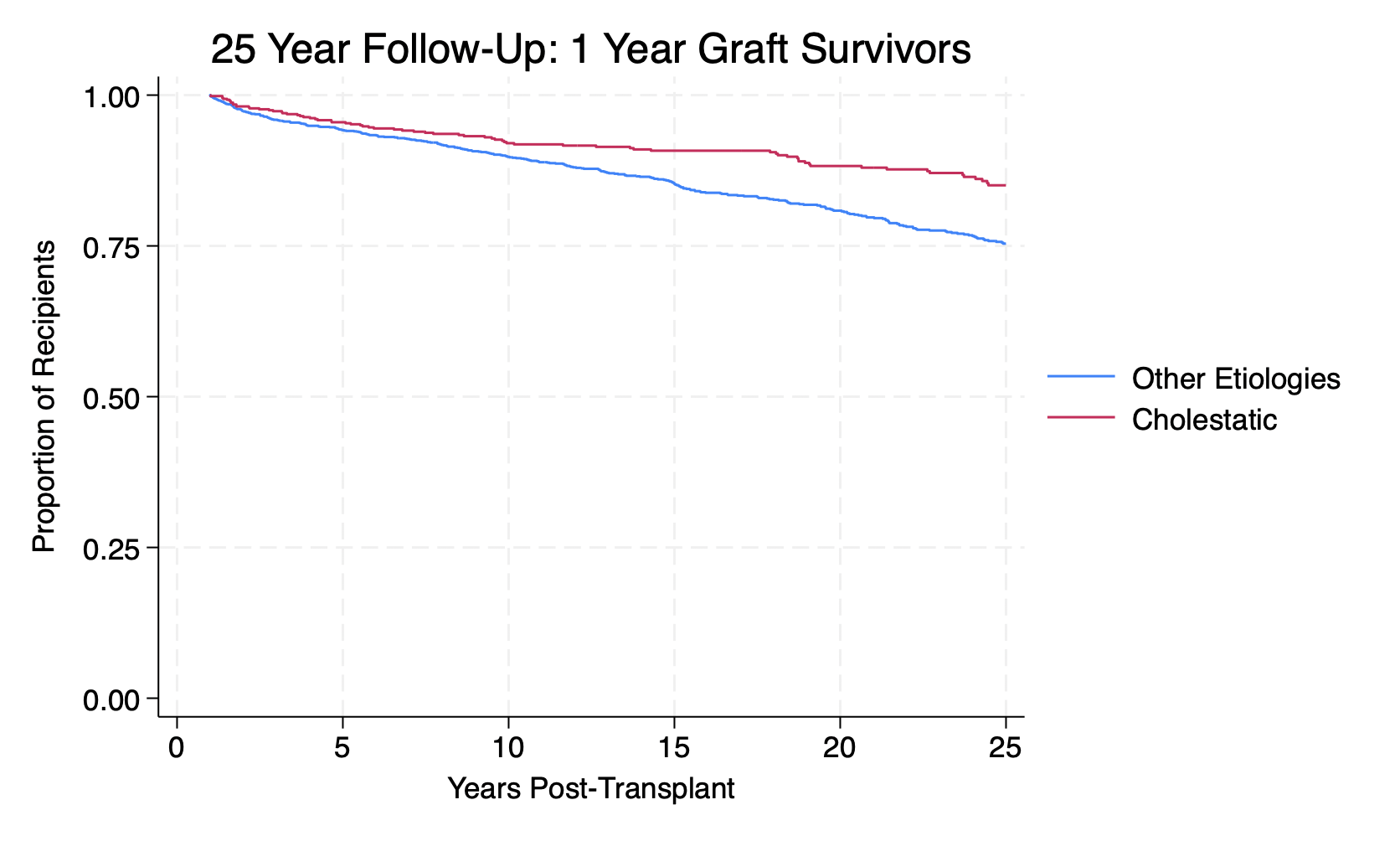
**

**Figure S2.** This figure illustrates a 25-year Kaplan-Meier Survival curve for cholestatic patients who survived at least one year.


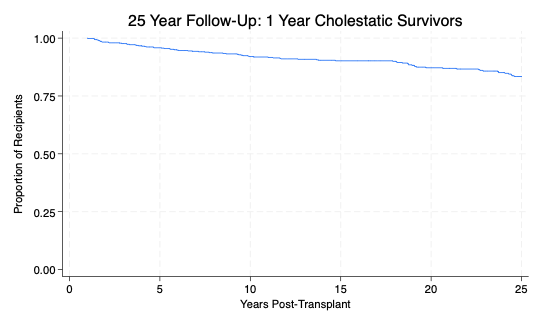


###

###

### 
